# ﻿New species and new records of the genus *Filatima* Busck, 1939 (Lepidoptera, Gelechiidae) from Central Asia

**DOI:** 10.3897/zookeys.1099.82530

**Published:** 2022-05-03

**Authors:** Oleksiy Bidzilya, Peter Huemer

**Affiliations:** 1 Institute for Evolutionary Ecology of the National Academy of Sciences of Ukraine, 37 Academician Lebedev str., 03143, Kiev, Ukraine Institute for Evolutionary Ecology of the National Academy of Sciences of Ukraine Kiev Ukraine; 2 Tiroler Landesmuseen Betriebsges.m.b.H., Sammlungs- und Forschungszentrum, Naturwissenschaftliche Sammlungen, Krajnc-Str. 1, 6060 Hall in Tirol, Austria Tiroler Landesmuseen Betriebsges.m.b.H. Hall in Tirol Austria

**Keywords:** Afghanistan, Gelechiinae, Iran, Pakistan, Palaearctic Region, Russia, systematics, taxonomy

## Abstract

Four new species of *Filatima* Busck, 1939 are described from Central Asia: *Filatimaarmata***sp. nov.** (Iran), *F.subarmata***sp. nov.** (Pakistan, Iran), *F.afghana***sp. nov.** (Afghanistan), and *F.karii***sp. nov.** (Tajikistan). The hitherto unknown female of *Filatimamulticornuta* Bidzilya & Nupponen, 2018 is described. Recorded to occur for the first time are *Filatimatextorella* (Chrétien, 1908) from North Macedonia and Turkey, *F.pallipalpella* (Snellen, 1884) from Kyrgyzstan, and *Filatimazagulajevi* Anikin & Piskunov, 1996 from Kazakhstan. *Filatimafontisella* Lvovsky & Piskunov, 1989 is removed from the list of Russian Gelechiidae due to re-identification of the only record as *F.multicornuta*. An annotated checklist of Palaearctic *Filatima* species is provided.

## ﻿Introduction

*Filatima* Busck, 1939 is a large genus of Holarctic Gelechiidae with the majority of species known from North America (Lee et al. 2009). The systematic position of the genus is still rather unclear. Both male and female genitalia are very peculiar and show no clear relation to other genera in the Gelechiidae. However, species in the genus share the feature of a deeply separated segment VIII into a free tergum and sternum, which is a putative synapomorphy for Gelechiinae (Hodges 1999). Within this subfamily the genus has been placed in the tribe Gelechiini provisionally near *Aroga* Busck, 1914 and *Athrips* Billberg, 1820 (Huemer and Karsholt 1999). Hodges (1999: 15) proposed and developed the argument that *Chionodes* Hübner, [1825], *Aroga*, and *Filatima* “comprise a closely related, highly speciose group”. Recently obtained results of molecular studies place the genus closest to *Aroga* and *Stegasta* Meyrick, 1904 (Karsholt et al. 2013), and these authors already stressed the need of increased taxon sampling.

Eight species of the European fauna were revised by Huemer and Karsholt (1999). Later two additional species were described, one from Romania (Kovács and Kovács 2001) and one from Spain (Corley 2014). Compared with the European fauna the Asian species remained poorly studied. By the end of the 20^th^ century only nine species had been recorded from Kyrgyzstan eastwards to the Amur region of Russia and Eastern China (Sattler 1968; Ivinskis and Piskunov 1989; Lvovsky and Piskunov 1989; Bidzilya et al. 1998). Recently new species were described from Southern Siberia and two new synonyms have been established (Bidzilya and Nupponen 2018). On the basis of these studies the genus currently comprises 57 Nearctic (Lee et al. 2009) and 19 Palaearctic species.

Here we provide descriptions of four new species from Central Asia, and also describe the hitherto unknown female of *F.multicornuta* Bidzilya & Nupponen, 2018. We also provide an annotated list of Palaearctic species of *Filatima* updated according to taxonomic changes proposed in the last few decades and new faunistic records.

## ﻿Materials and methods

Male and female genitalia were dissected and prepared using standard methods for the Gelechiidae (Huemer and Karsholt 2010). Male genitalia were spread implementing the unrolling technique described by Pitkin (1986) and Huemer (1988). The descriptive terminology of the genitalia structures follows Huemer and Karsholt (1999); the order of species in the checklist is alphabetical. Pinned specimens were photographed with a Canon EOS 5DSR DSLR camera attached to an Olympus SZX12 stereomicroscope. Slide-mounted genitalia were photographed with a Canon EOS 600D DSLR camera mounted on an Olympus U-CTR30-2 trinocular head mounted on a Carl Zeiss compound microscope. For each photograph, sets of 10–20 images were taken at different focal planes and focused-stacked using Helicon Focus 6 with the final image edited in Adobe Photoshop CS5.

### ﻿Abbreviations of collections

**NHMB** Hungarian Natural History Museum, Budapest, Hungary

**NHMV** Naturhistorisches Museum, Vienna, Austria

**NHMUK** Natural History Museum, London, U.K.

**NUPP** Research Collection of Kari & Timo Nupponen, Espoo, Finland

**SMNK** Staatliches Museum für Naturkunde Karlsruhe, Germany

**TLMF** Tiroler Landesmuseum Ferdinandeum, Innsbruck, Austria

**ZIN** Zoological Institute, Russian Academy of Sciences, Sankt-Petersburg, Russia

**ZMKU** Zoological Museum, Kyiv Taras Shevchenko National University, Kyiv, Ukraine

**ZMUC** Zoological Museum, Natural History Museum of Denmark, Copenhagen, Denmark

### ﻿Other abbreviations

**HT** holotype

**PT** paratype

**OB** Oleksiy Bidzilya

## ﻿Results

### ﻿Taxonomic account

#### ﻿ Filatima armata sp. nov.

Taxon classificationAnimaliaLepidopteraGelechiidae

CB27BDB3-780A-5782-8A4F-1366F1ACF154

http://zoobank.org/C0D30913-82AA-471D-ABAE-8C83CAF79538

Figs 1 , 5 , 9–18 , 19–21

##### Material examined.

***Holotype*** [Iran] • ♂; Khusestan, Yassudi, Sisakht; 2250 m; 13–14 Jun 1972; [genitalia slide number] 73/17, O. Bidzilya; G. Ebert and H. Falkner leg; SMNK. ***Paratypes*** [Iran] • 1 ♂, 2 ♀♀; same collection data as for holotype; [genitalia slide number] 74/17♀, O. Bidzilya • 1 ♂; Khusestan, Yassudi, Sisakht; 2250 m; 15–18 Jun 1975; G. Ebert and H. Falkner leg. • 1 ♀; Khusestan, 15 km SE Yassudi; 2050 m; 15 Jun 1972; [genitalia slide number] 6/18, O. Bidzilya; G. Ebert and H. Falkner leg. • 5 ♀♀; Khusestan, 30 km S Yassudi, Kuschk; 2220 m; 12 Jun 1972 [genitalia slide number] 73/17, O. Bidzilya; G. Ebert and H. Falkner leg. • 1 ♂; Fars, 50 km NW Ardekan, Tange Surkh; 2250 m; 16 Jun 1972 [genitalia slide number] 5/18, O. Bidzilya; G. Ebert and H. Falkner leg. • 4 ♀♀; Fars, 50 km NW Ardekan, Tange Surkh; 2250 m; 12–15 Jun 1975; [genitalia slide number] 46/22, O. Bidzilya; G. Ebert and H. Falkner leg. • 1 ♀; Fars, Daschte Ardian, Kotal-Pirehsan; 2000 m; 18 Jun 1972 [genitalia slide number] 77/17, O. Bidzilya; G. Ebert and H. Falkner leg. • 1 ♂, 1 ♀; Strasse Shiraz-Kazeru, Imam Sade; 1200 m; 3 Jun 1969; H. Amsel leg. • 2 ♂♂; Sineh Safid, Fars, FF. 57; c. 6500 ft; 19 May 1950 [genitalia slide number] 78/17; 24/18, O. Bidzilya; E. P. Wiltshire leg. • 1 ♀; Baloutchistan, Kouh i Taftan (Khach); 2500 m; 28 Jun1938; F. Brandt leg. • 1 ♂; Elburs Gebirge, Keredj; [day? month?]1936; F. Brandt leg. • 1 ♂; Fars, Strasse Chiraz-Kazeroun, Fort Sine-sefid; 2200 m; 28 Jun 1938; F. Brandt leg. • 1 ♂; Fars, Strasse Ardekan-Talochosroe, Comé; 2600 m; 29 Jul 1937; F. Brandt leg.; all SMNK • 3 ♂; Berge O Kasri Schirin; 24 May 1963; [genitalia slide number] MV 15.338♂, P. Huemer; F. Kasy and E. Vartian leg. • 1 ♂, 1 ♀; 65 km W Shiraz; 16 Apr 1970; [♂ genitalia in glycerin]; Exp. Mus. Vind.; all NHMV • 1 ♂; Khorasan, Qucahn; 19 May 2010; G. Petrányi and P. Hentschel leg. • 1 ♂; Kars, Ardekan, Sepidan; 8–11 May 2010; G. Petrányi and P. Hentschel leg. • 1 ♂; Hamedan, Nehavand, 13 May 2010; G. Petrányi and P. Hentschel leg.; all ZMUC.

**Figures 1–8.**
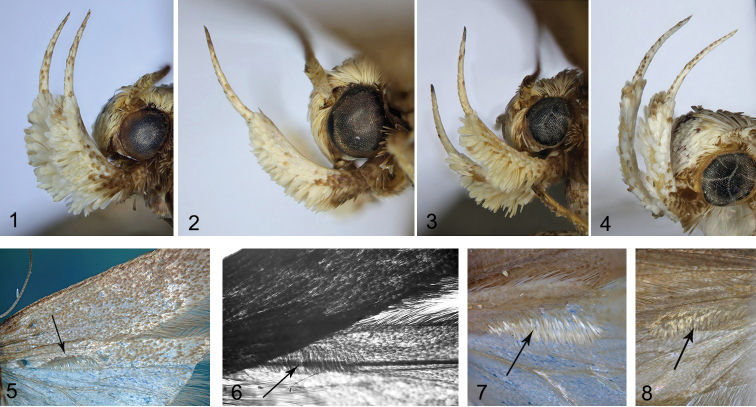
*Filatima* spp., details of external morphology **1–4** head, lateral view **1***F.armata* sp. nov., PT, 50 km NW Ardekan, Tange Surkh **2***F.subarmata* sp. nov., PT, Quetta **3***F.afghana* sp. nov., PT, Kabul **4***F.karii* sp. nov., HT**5–8** male hindwing, underside (arrow indicates row of caudally directed scales on R5) **5***F.armata* sp. nov., HT**6, 7***F.subarmata* sp. nov., PT, 70 km S. v. Teheran **6** in transmitted light **7** in reflected light **8***F.afghana* sp. nov., PT, Sarobi.

##### Diagnosis.

**Figures 9–18.**
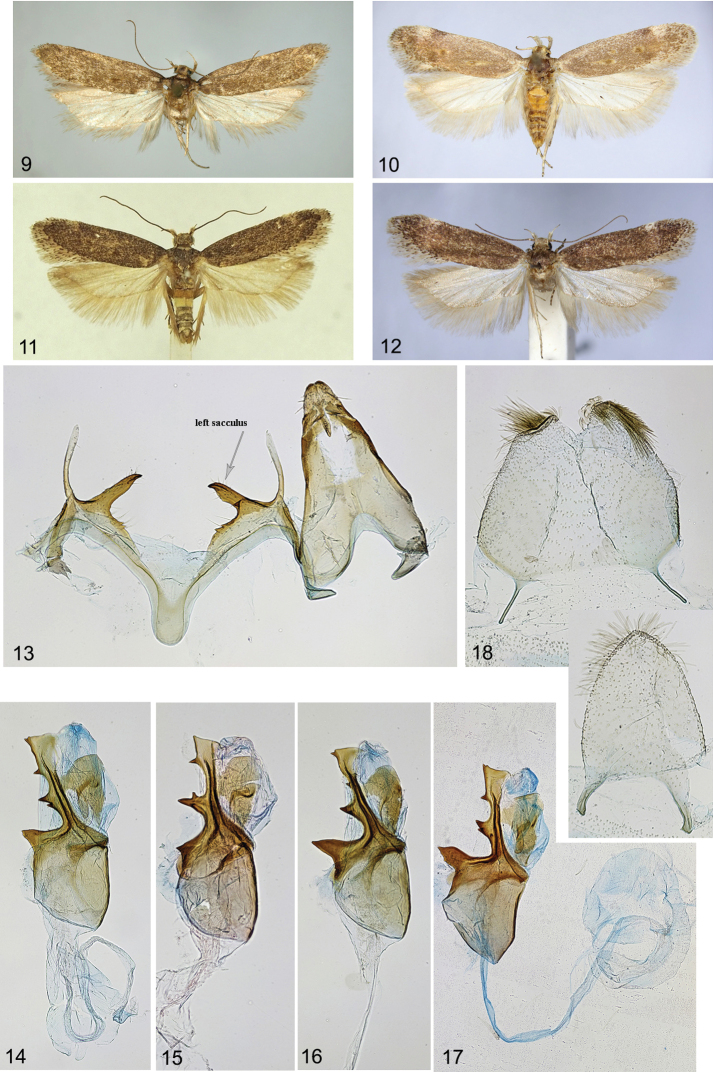
*Filatimaarmata* sp. nov. **9–12** adults **9** holotype **10** paratype, female, S Iran (gen. slide 46/22, O. Bidzilya) **11** paratype, male, S Iran **12** paratype, female, Pakistan (gen. slide 39/22, O. Bidzilya) **13** male genitalia (gen. slide 24/18, OB) **14–17** phallus, Iran **14** gen. slide 24/18, OB**15** gen. slide 2/18, OB**16** gen. slide 5/18, OB. **17**HT, gen. slide 73/17, OB**18** male segment VIII, gen. slide 24/18, OB.

**Figures 19–21.**
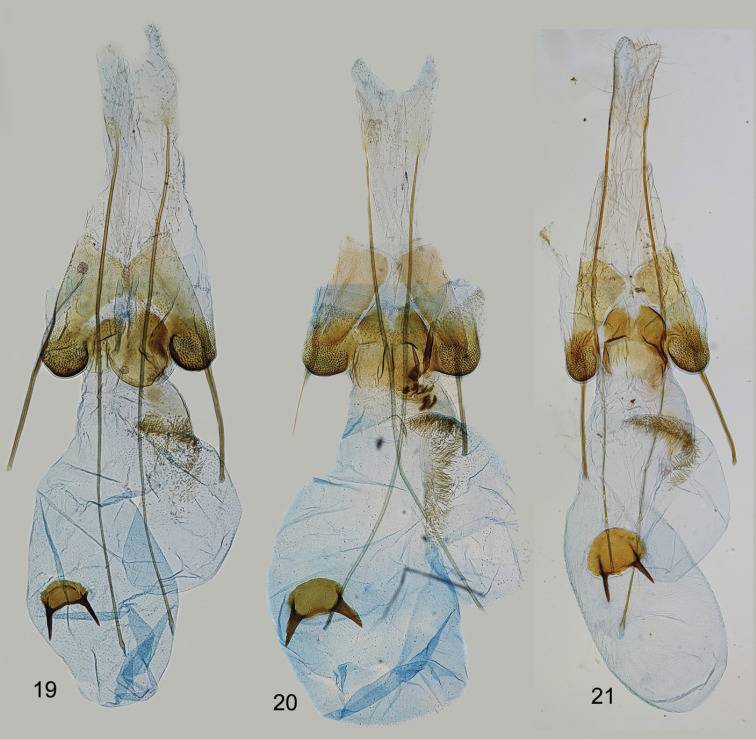
*Filatimaarmata* sp. nov., female genitalia **19** gen. slide 6/18, OB**20** gen. slide 74/17, OB**21** gen. slide 46/22, OB.

The new species has the elongate uniformly brown forewings usually with markings (Figs 9–12) which are typical for *Filatima*, *Chionodes* and other nearby taxa in Gelechiinae. It is similar externally to *F.textorella* (Chrétien, 1908) and *F.transsilvanella* Kovács & Kovács, 2001, but the first species does not have a row of caudally directed scales to 1/2 of R5 on the underside of the male hindwing, which is present in the male of *F.transsilvanella* and *F.armata* sp. nov. (Fig. 5). There are no reliable external differences for *F.subarmata* sp. nov. The male genitalia (Fig. 13) are distinctive in having weakly asymmetrical sacculi with a small tooth at the base of the left one; despite some variation, the phallus (Figs 14–17) is also very peculiar having a strongly sclerotised longitudinal ribbon with three large and several small lateral thorns and a sclerotised plate in the vesica. *Filatimatranssilvanella* differs in the longer uncus, the absence of a tooth on the right sacculus and the phallus having smaller thorns and without a sclerotised plate in the vesica. The female genitalia (Figs 19–21) are identifiable from the ribbon of long, needle-shaped spines in the bulla seminalis in combination with broadly rounded lateral sclerites, and a short sub-rectangular medial sclerite with an emarginated posterior margin. Among Palaearctic *Filatima* species the bulla seminalis is known in *F.transsilvanella*, *F.pallipalpella* (Snellen, 1884), and *F.afghana* sp. nov. The first species has a rounded and short bulla seminalis (Kovács and Kovács 2001; Junnilainen et al. 2010), whereas *F.pallipalpella* has an elongate one with short spines. *Filatimaafghana* sp. nov. like *F.armata* sp. nov. has a ribbon of needle-shaped spines, but differs in the narrower and inwardly curved lateral sclerites.

##### Description.

(Figs 1, 5, 9–12). Wingspan 15.0–22.0 mm. Head light brown, frons paler, greyish white, labial palpus (Fig. 1) recurved, segment 2 greyish white, dark brown at base, upperside brown, underside with brush of modified scales, segment 3 light brown with a few dark scales, antennal scape and flagellum brown; thorax, tegulae and forewing (Figs 9–12) uniformly brown, fold mixed with ochreous, ochreous brown spot in fold and in cell in some specimens, diffuse white spot at 3/4 of costal margin, cilia tipped grey-brown; hindwing grey, row of caudally directed scales to 1/2 of R5 underside in male (Fig. 5); abdominal terga I–IV yellow, remaining terga grey.

***Male genitalia*** (Figs 13–18). Tergum VIII tongue-shaped, with long, narrow anterolateral arms; sternum VIII rounded to sub-trapezoidal, posterior margin with paired patch of hairs and with short medial emargination, anterolateral arms long and narrow (Fig. 18). Uncus sub-trapezoidal, weakly narrowed apically, posterior margin weakly rounded, with short triangular medial incision, laterally covered with strong setae; gnathos slightly longer than uncus, medial sclerite weakly curved, distally weakly serrate on dorsal surface; tegumen sub-triangular, gradually narrowed distally, anteromedial incision reaching to ~ 1/3 of its length; valva short and slender, subapex weakly broadened; sacculus inwardly turned, ~ 2/3 as long and 3 × as broad as valva, left sacculus broader basally and shorter than left one, with small basal tooth; vinculum with broad and deep sub-triangular medial emargination, weakly serrate posteriorly; saccus 1.5–2 × longer than broad, sub-rectangular, apex rounded; phallus as long as tegumen, swollen at base, distal 2/3 with sclerotised ribbon along the left side and four strong lateral thorns: basal one longest, triangular, medial one shortest, paired, subapical thorn very short, and apical one largest, subtriangular, vesica with large irregular sclerotised plate, bulbus ejaculatorius long, coiled.

**Variation.** Adults vary in size from 15.0 to 22.0 mm in wingspan. Valva, saccus, and thorns of the phallus vary in length.

***Female genitalia*.** (Figs 19–21). Papillae anales sub-ovate, elongated, setose; apophyses posteriores extending the length of corpus bursae, apophyses anteriores shorter than segment VIII, straight; sternum VIII longer than broad, sub-rectangular, weakly narrowed posteriorly, sub-genital plates weakly broadened and joined posteromedially, medial area membranous, mainly covered with fine microtrichia medially and anteriorly, lateral sub-ostial sclerite densely covered with short teeth, broad, rounded, medial sub-ostial sclerite sub-rectangular to rounded with posteromedial emargination; antrum half the length of apophyses anteriores, with strongly sclerotised edge in anterior part; ductus bursae short, broad, with indistinct transition to corpus bursae, with bulla seminalis arising from the right side and extending to 1/2–2/3 length of corpus bursae, with ribbon of long and narrow needle-shaped spines extending from ductus bursae to base of bulla seminalis corpus bursae broadly rounded; signum plate sub-ovate with paired long, narrow, acute sclerites directed anteriorly.

##### Biology.

The adults have been collected from mid-April to late July at altitudes between 1200 and 2600 m.

##### Distribution.

Iran.

##### Etymology.

The name of the new species is derived from the Latin *armatus* meaning armed warrior, and refers to the strongly sclerotised phallus armed with strong thorns.

#### ﻿ Filatima subarmata sp. nov.

Taxon classificationAnimaliaLepidopteraGelechiidae

22DAE1C6-07EF-555E-8E35-F26A625BA401

http://zoobank.org/4804423B-3B9F-4FD9-BE65-7565AA16EE6D

Figs 2 , 6 , 7 , 22–29

##### Material examined.

***Holotype*** [Pakistan] • ♂; 80 km NW v. Quetta; 2100 m; 15 May 1965; [genitalia slide number] 45/22, O. Bidzilya; F. Kasy and E. Vartian leg.; NHMV. ***Paratypes*** • 1 ♂; same collection data as for holotype; [genitalia slide number] 34/22, O. Bidzilya; [Iran] • 2 ♂♂; 70 km S. v. Teheran; 1300 m; 5 May 1965; [genitalia slide number] 54/22, O. Bidzilya; F. Kasy and E. Vartian leg.; all NHMV; • 1 ♂, same data as for proceeding but ex coll. Glaser [genitalia slide number] 91/18, O. Bidzilya; SMNK.

##### Diagnosis.

The new species shows a close relationship with the previous one in respect of the male genitalia and external appearance. However, the male genitalia (Figs 24, 25) of *F.subarmata* sp. nov. differ in the shorter and broader left sacculus and the broader right sacculus. Additionally, the basal thorn of the phallus is shorter, the medial thorn is elongate and apically bifurcate rather than triangular as in *F.armata* sp. nov. and a small subapical thorn is absent in *F.subarmata* sp. nov. (Figs 26–29). We observed also differences in the shape of the saccus which is slightly longer and narrower in *F.subarmata* sp. nov. We did not find reliable differences in the external appearance between *F.subarmata* sp. nov. and *F.armata* sp. nov.

##### Description.

**Figures 22–29.**
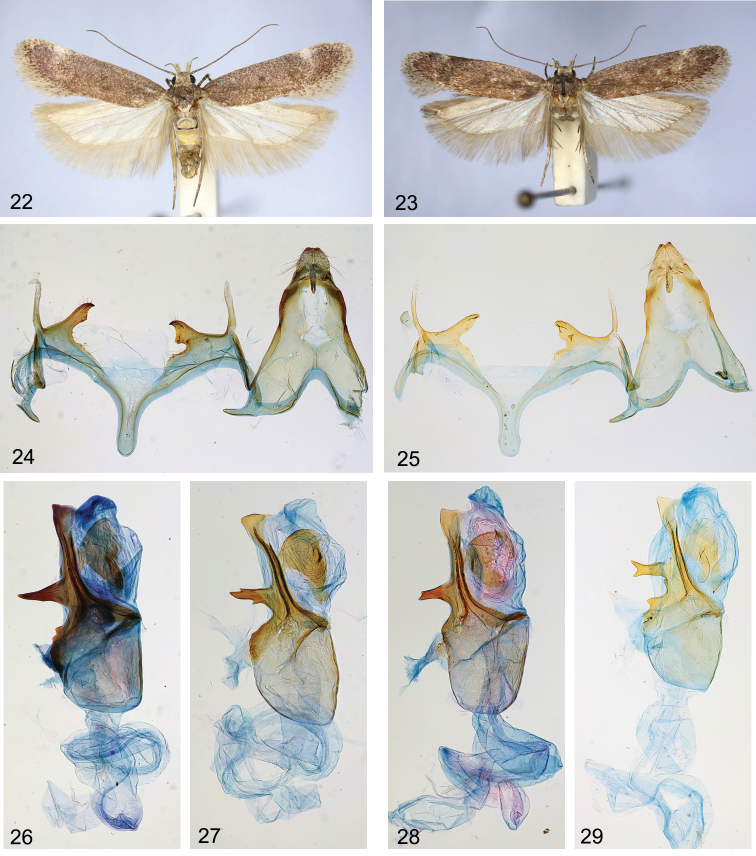
*Filatimasubarmata* sp. nov. **22, 23** adult **22**HT**23**PT, Iran (gen. slide 54/22, OB) **24, 25** male genitalia **24**HT**25**PT, Iran (gen. slide 91/18, OB) **26–29** Phallus **26**HT**27**PT, Iran (gen. slide 54/22, OB) **28**PT, Pakistan (gen. slide 34/22, OB) **29**PT, Iran (gen. slide 91/18, OB).

(Figs 2, 6, 7, 22, 23). Wingspan 18.1–19.1 mm. Head covered with grey brown-tipped scales, frons white to pale, labial palpus (Fig. 2) recurved, far protruded over the head, yellowish white, segment 2 with brown base and a few light brown scales on inner surface mainly, on underside with brush of modified scales, segment 3 approximately 2/3 length and 1/3 width of segment 2, mottled with brown; scape brown with a few grey scales at apex, antennal flagellomeres brown with indistinct grey rings; thorax and tegulae brown mixed with grey; forewing (Figs 22, 23) brown rarely mixed with grey, three diffuse indistinct dark, ochreous-brown spots in cell, fold with ochreous brown suffusion, white costal spot at 3/4, subapical pale narrow transverse fascia weakly indicated, cilia tipped grey-brown; hindwing grey, with darkened veins, margins and apex, row of caudally directed scales to 1/2 of R5 underside (Figs 6, 7), cilia grey.

***Male genitalia*** (Figs 24–29). Tergum VIII tongue-shaped, with long, narrow anterolateral arms; sternum VIII rounded to sub-trapezoidal, posterior margin with paired patch of hairs and with short medial emargination, anterolateral arms long and narrow. Uncus sub-trapezoidal, weakly narrowed apically, posterior margin weakly rounded, with short triangular medial incision, laterally covered with strong setae; gnathos slightly longer than uncus, medial sclerite weakly curved, distally weakly serrate on dorsal surface; tegumen sub-triangular, gradually narrowed distally, anteromedial incision reaching to ~ 1/3 of its length; valva short and very slender, bluntly acute; sacculus curved medially, ~ 1/2 length and 4 × as broad as valva, the left sacculus broader and shorter than the right one, with small basal tooth; vinculum with broad and deep sub-triangular medial emargination, weakly serrated posteriorly; saccus 2 × longer than broad, sub-rectangular, apex rounded; phallus as long as tegumen, swollen at base, distal 2/3 with a sclerotised ribbon along the left side with four lateral thorns: two basal thorns are short, triangular, the medial thorn is the longest, slender, bifurcated apically except the HT (Fig. 26), and the apical one is the broadest, subtriangular, vesica with large irregular sclerotised plate, bulbus ejaculatorius long, coiled.

***Female genitalia*.** Unknown.

##### Biology.

Adults have been collected in May at altitudes of 2100 m in Pakistan and 1300 m in Iran.

##### Distribution.

Pakistan, Iran.

##### Etymology.

The specific name reflects the relationship of the species to *F.armata* sp. nov.

#### ﻿ Filatima afghana sp. nov.

Taxon classificationAnimaliaLepidopteraGelechiidae

8AC6919E-B59D-5E74-BE7E-C79DA0E335EA

http://zoobank.org/872CE8C8-EEC4-437D-B3F4-82BFE0B03DD3

Figs 3 , 8 , 30–36 , 37–39

##### Material examined.

***Holotype*** [Afghanistan] • ♀; Pol-i-Charchi, 18 km östl. Kabul; 1700 m, 25 Jun –3 Jul 1966; H. Amsel leg.; SMNK. ***Paratypes*** [Afghanistan] • 1 ♀; Safed Koh, S Seite Kotkai; 2350 m; 19–23 Jun 1966; H. Amsel leg. • 1 ♂; Sarobi, 1100 m; 17 Aug 1961; [genitalia slide number] 47/17, O. Bidzilya; G. Ebert leg. • 1 ♂, 1 ♀; Sarobi, 1100 m; 13 Aug 1961; [genitalia slide number] Am. 1756♂, D. Povolný; 45/17♀, O. Bidzilya; G. Ebert leg. • 2 ♀♀; Arghandab-Damm, 35 km ndl. Kandahar; 1150 m; 23/27 May 1961; [genitalia slide number] Am. 1761♀, D. Povolný; 3/18, O. Bidzilya; G. Ebert leg. • 1 ♂, 2 ♀♀; Herat; 970 m; 5 May 1956; [genitalia slide number] Am. 1720♂, D. Povolný; 25/18♀, O. Bidzilya; H. Amsel leg.; all SMNK • 3 ♂♂, 2 ♀♀; 40 km SW v. Kabul; 2300 m; 29 Jun 1965; [genitalia slide number] MV 16.509♀, MV 15.340♂, MV 16.510♂, MV 16.512♂, P. Huemer; 57/22♀, O. Bidzilya; F. Kasy and E. Vartian leg. • 1 ♀; 80 km NO v. Kandahar; 27 Jun 1963; F. Kasy and E. Vartian leg.; all NHMV; [Pakistan] • 1 ♀; 80 km NW v, Quetta; 2100 m; 15 May 1965; [genitalia slide number] 39/22♀, O. Bidzilya; F. Kasy and E. Vartian leg.; all NHMV.

##### Diagnosis.

**Figures 30–36.**
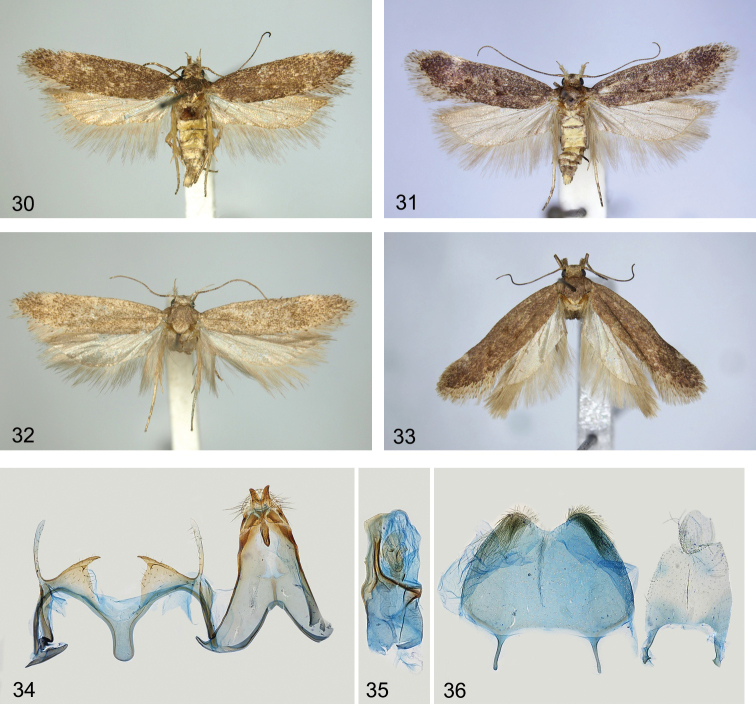
*Filatimaafghana* sp. nov. **30–33** adults **30**HT**31**PT, female (gen. slide 57/22, OB) **32**PT, female (gen. slide 25/18, OB) **33**PT, female (gen. slide 91/18, OB) **34–36** male genitalia and abdominal segment VIII (gen. slide 47/17, OB) **34** unrolling **35** phallus **36** abdominal segment VIII.

The new species is rather uniformly dark brown (Figs 30–33), darker than *F.armata* sp. nov. and *F.subarmata* sp. nov., with indistinct markings. It is very similar externally to those two species, but on average it has a smaller wingspan, is darker, and has a paler, white rather than greyish white, head and labial palpus. The apically bifurcate uncus, short and narrow sacculus with a basal tooth (Fig. 34), and phallus with longitudinal sclerotised ribbon and sclerotised plate of the vesica (Fig. 35) are characteristic in the male genitalia. *Filatimatranssilvanella* differs in the longer uncus that is not divided apically and the longer and broader sacculus without a basal tooth. The female genitalia are recognisable by the ribbon of long needle-shaped spines in the bulla seminalis in combination with narrow inwardly curved lateral sclerites. *Filatimatranssilvanella* differs in the rounded rather than elongate bulla seminalis, longer apophyses anteriores and the lateral sclerite that is not turned inwards.

##### Description.

(Figs 3, 8, 30–33). Wingspan 13.0–17.3 mm. Head pale white, neck distinctly mottled with brown, labial palpus recurved, segment 2 creamy white, dark brown at base, underside with brush of modified scales, segment 3 slender, 1/3 width and approx. as long as segment 2, brown, upper side white, antennal scape and flagellum brown (Fig. 3); thorax, tegulae and forewing uniformly brown, fold mixed with ochreous, ochreous brown spot in fold and in cell in some specimens, diffuse white spot on 3/4 of costal margin, cilia tipped grey-brown; hindwing grey, row of caudally directed scales to 1/2 of R5 underside in male (Fig. 8); abdominal terga I–IV yellow, remaining terga grey.

***Male genitalia*** (Figs 34–36). Tergum VIII tongue-shaped, with long, narrow anterolateral arms; sternum VIII rounded to sub-trapezoidal, posterior margin with paired patch of hairs and with shallow medial emargination, anteromedial arms long and narrow (Fig. 36). Uncus deeply divided posteromedially into digitate lobes that are weakly narrowed apically and covered with strong setae laterally; gnathos approx. as long as uncus, medial sclerite weakly curved, dorsal surface with several folds; tegumen sub-triangular, gradually narrowed distally, anteromedial incision reaching to ~ 1/3 of its length; valva slender, apex weakly broadened; sacculus short, narrow, acute, inwardly turned, with basal tooth; vinculum with broad and deep U-shaped medial emargination; saccus 2 × longer than broad, sub-rectangular, apex weakly rounded; phallus slightly shorter than tegumen, nearly of equal width, weakly narrowed at base, distal 2/3 with a sclerotised ribbon along the left side, with two small teeth in one specimen and without them in other specimens, vesica with large irregular sclerotised plate, bulbus ejaculatorius long, coiled.

**Figures 37–39.**
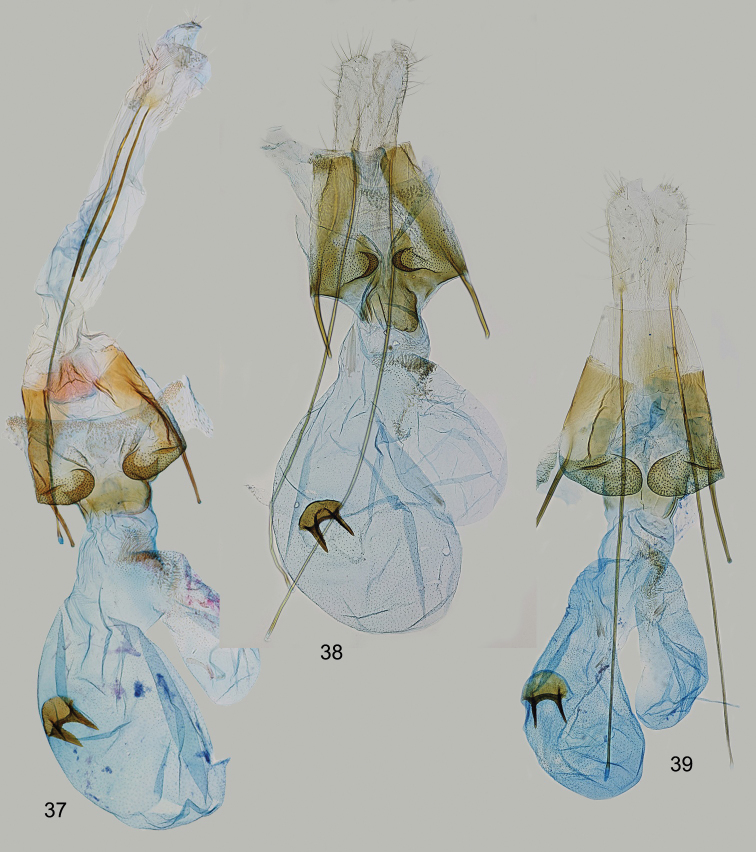
*Filatimaafghana* sp. nov., female genitalia **37** gen. slide 57/22, OB**38** gen. slide 25/18, OB**39** gen. slide 45/17, OB.

**Variation**. Left sacculus is broader than the right one in one specimen; the basal tooth of the sacculus among specimens varies in size.

***Female genitalia*** (Figs 37–39). Papillae anales sub-ovate, elongate, setose; apophyses posteriores 1.3–1.5 as long as length of bursae copulatrix; apophyses anteriores shorter than segment VIII, straight; sternum VIII longer than broad, sub-rectangular, weakly narrowed posteriorly, with large, weakly sclerotised posteromedial plate, subgenital plates 1/4–1/3 width of segment VIII, medial 1/3–2/3 membranous, mainly covered with fine microtrichia medially and anteriorly, lateral sub-ostial sclerite densely covered with short teeth, elongated, turned inwards, rounded, more strongly sclerotised and edged medially; medial sub-ostial sclerite weakly sclerotised, sub-rectangular to rounded with posteromedial emargination; antrum subquadrate, shorter than apophyses anteriores; ductus bursae short, broad with indistinct transition to corpus bursae, with broad bulla seminalis arising from right side and extending to 1/2 –2/3 length of corpus bursae, with ribbon of long and narrow needle-shaped spines extending from ductus bursae to base of bulla seminalis, ductus seminalis arising from anterior part of bulla seminalis; corpus bursae broadly rounded; signum plate sub-ovate, strongly sclerotised and weakly serrate anteriorly with pair of lateral long, narrow acute sclerites directed anteriorly.

##### Variation.

The shape of the posteromedial plate varies from sub-triangular to sub-rectangular; lateral sub-ostial sclerite varies in width from elongate to broadly rounded, usually with distinct sclerotisation in medial 1/4, but is uniformly sclerotised in one specimen; apophyses anteriores vary in length from as long as, to shorter than segment VIII.

##### Biology.

The adults have been collected from early May to mid-August at altitudes between 970 and 2350 m.

##### Distribution.

Afghanistan, Pakistan.

##### Etymology.

The specific name reflects the distribution of this new species in Afghanistan.

#### ﻿ Filatima karii sp. nov.

Taxon classificationAnimaliaLepidopteraGelechiidae

79F7E78D-7EA5-50EC-9099-9550EFECC8DA

http://zoobank.org/5322FBFF-6C65-47A4-9BC8-E64BEAC3C2FD

Figs 4 , 40–43

##### Material examined.

***Holotype*** [Tajikistan] • ♂; W-Pamir mts, 37°00'55"N, 72°34'32"E, Pijanj/Pamir River by Zugvand village; 2810 m; 25 Jul 2013; [genitalia slide number] 152/16, O. Bidzilya; K. Nupponen & R. Haverinen leg.; NUPP.

##### Diagnosis.

**Figures 40–43.**
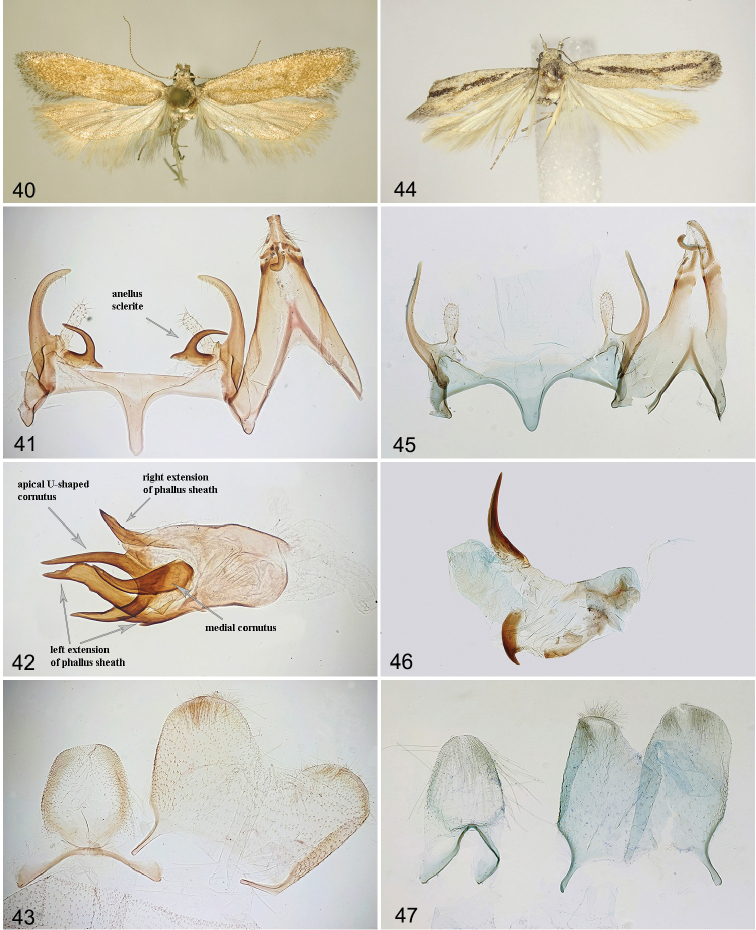
*Filatimakarii* sp. nov., holotype **40** adult **41** male genitalia, unrolling **42** phallus **43** segment VIII **44–47***Filatimanigrimediella* Bidz., holotype **44** adult **45** male genitalia, unrolling (gen. slide 109/18, OB) **46** phallus (gen. slide 109/18, OB) **47** segment VIII (gen. slide 109/18, OB).

Externally this new species is recognised by the light brown forewing with the costal margin distinctly mottled with black (Fig. 40). *Filatimafontisella* Lvovsky & Piskunov, 1989 from Mongolia shares with *Filatimakarii* sp. nov. the absence of row of scales on the ventral surface of the male hindwings and somewhat similar forewing pattern. However, in *F.fontisella* forewing is lighter, pale yellow, and the wingspan is smaller (11–15 mm contrary to 17.2 mm in *F.karii* sp. nov.). The male genitalia (Figs 41, 42) resemble those of *F.fontisella*, *F.ukrainica* Piskunov, 1971, and *F.multicornuta*, all with well-developed horn-shaped anellus sclerites. Apical U-shaped (V-shaped in above species) cornutus and very broad left extension of the phallus sheath are characteristic for the new species.

##### Description.

(Figs 4, 40). Wingspan 17.2 mm. Head covered with pale white, brown-tipped scales, frons white, labial palpus recurved, segment 2 white, dark brown at base, underside with brush of modified white scales and few brown scales at apex, segment 3 slender, 1/3 width and ~ 2/3 length of segment 2, white mixed with light brown, antennal scape white densely mixed with brown, flagellum ringed white and brown (Fig. 4); thorax and tegulae slightly darker than neck, brown mottled with pale white; forewing light brown, slightly darker in distal 2/3, diffuse dark brown spots in mid-wing at 1/3 and 2/3, fold slightly darker than adjacent area of forewing, costal margin and base with distinct black suffusion, cilia pale white to light brown, with distinct brown tips; hindwing grey in basal half and darker, light brown in distal half, veins distinct, mottled with dark brown.

***Male genitalia*** (Figs 41–43). Tergum VIII egg-shaped, with distinct moderately broad anterolateral arms, anterior margin sclerotised; sternum VIII subtrapezoidal, posterolateral corners rounded, posteromedial emargination broad, anterolateral arms long and narrow (Fig. 43). Uncus basally as broad as long, narrowed to 3/4 length, apically weakly widened, posterior margin straight, laterally covered with strong setae; gnathos approx. length of uncus, apical 1/3 of medial sclerite curved at right angle, weakly broadened; tegumen elongated, sub-triangular, gradually narrowed distally, anteromedial incision reaching to ~ 1/2 of its length; valva moderately broad, gradually tapered to a bluntly pointed apex, gradually curved, extending to apex of gnathos; sacculus membranous, finger-shaped, of even width and apex bluntly rounded, 1/2 length of valva; sclerites of anellus symmetrical, with stout base and long horn-shaped outwardly turned distal sclerite, as long as sacculus; vinculum short, band-shaped, saccus weakly narrowed towards rounded apex, slightly extending beyond anterior projection of pedunculus; phallus slightly shorter than tegumen, weakly narrowed at base, with medial horn-shaped cornutus and apical U-shaped cornutus with its right process slightly longer than left process; additionally, there are two lateral extensions of the phallus sheath: the left one is long and broad with two basal teeth on left side, and the right one is short, narrow; bulbus ejaculatorius short.

***Female genitalia*.** Unknown.

##### Biology.

The holotype was collected in late July at an altitude of c. 2800 m. The collecting site is the edge between a steep rocky slope and riverside sand dunes with plenty of *Salix* (see Bidzilya et al. 2019: 125, fig. 43).

##### Distribution.

Tajikistan.

##### Etymology.

We dedicate this species to the late Kari Nupponen, leading specialist on the Scythrididae, outstanding collector, and a wonderful friend who passed away much too early.

#### ﻿ Filatima multicornuta

Taxon classificationAnimaliaLepidopteraGelechiidae

Bidzilya & Nupponen, 2018

189905BA-A0F6-5317-B74F-878AA8CEA0C1

Figs 48 , 49

Filatima multicornuta Bidzilya & Nupponen, 2018: 395

##### Material examined.

[Mongolia] • 1 ♂, 1 ♀; Central aimak, 12 km S von Somon Bajanbaraat; 1380 m; 8 Jun 1967; [genitalia slide number] 214/20♂, 215/20♀, O. Bidzilya; Exp. Dr. Z. Kaszab, 1967, Nr. 776 • 1 ♂, 1 ♀; Bajanchongor aimak, 8 km S von Somon Zinst; 1400 m; 25 Jun 1964; [genitalia slide number] 49/22♂, 50/22♀, O. Bidzilya; Exp. Dr. Z. Kaszab, 1964, Nr. 198 • 1 ♀; Gobi Altaj aimak, NW Ecke des Chasat chajrchan ul Gebirge, 2 km NW von Somon Bičigt. 1900 m; 14 Jul 1966; [genitalia slide number] 62/22♀, O. Bidzilya; Exp. Dr. Z. Kaszab, 1966, Nr. 688; all NHMB.

**Figures 48–50.**
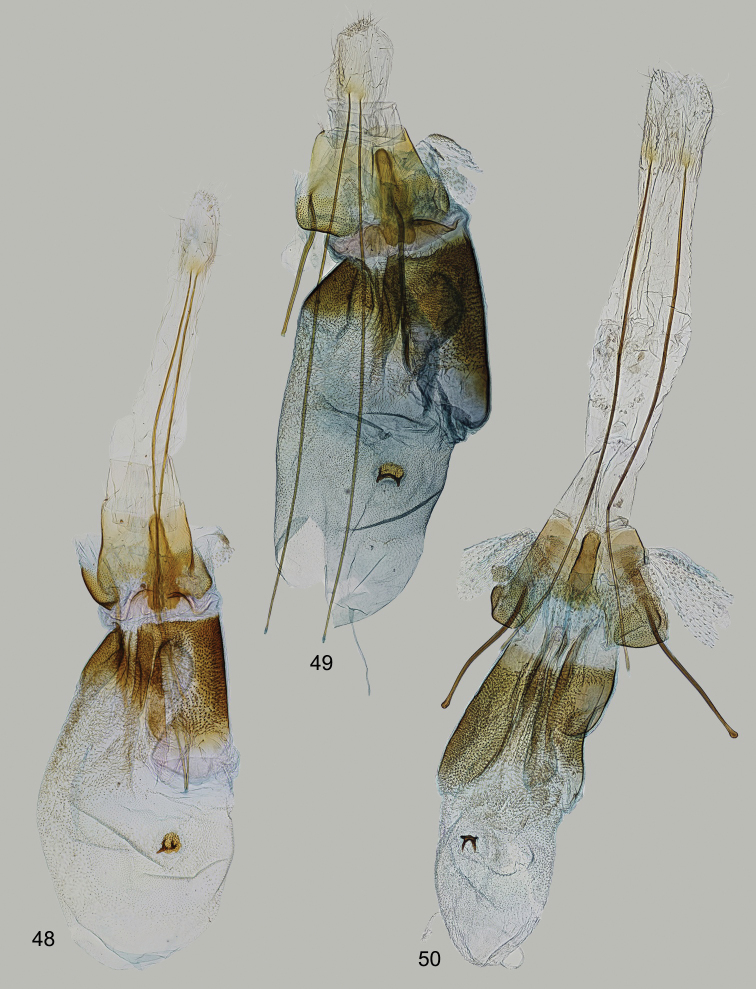
*Filatima* spp., female genitalia **48, 49***Filatimamulticornuta* Bidz. & Nupp., Mongolia. **48** Gen. slide 50/22, OB 49 gen. slide 215/20, OB**50***F.zagulajevi* Anikin & Piskunov, Kazakhstan, gen. slide 15/18, OB.

The species has been recently described from four males from Tuva Republic of Russia and Mongolian Altai. Our study of additional material from Mongolia resulted in the discovery of the hitherto unknown female which is described below.

***Female genitalia*** (Figs 48, 49). Papillae anales sub-ovate, densely covered with short setae; apophyses anteriores 4 × as long as apophyses posteriores; segment VIII subrectangular, slightly longer than broad; sternum VIII with posterior margin weakly emarginated, evenly sclerotised, with rounded sclerites covered with minute thorns at base of apophyses anteriores; medial sclerite narrow, cone-shaped, extending to the anterior margin of sternum VIII; ductus bursae short and broad, with indistinct transition to corpus bursae, numerous dense and strong needle-shaped spines do not extend so far anteriorly as on the right side, more delicate and less dense hair-like spines from 1/3 to 1/2 length in left side, several longitudinal overlapping folds extending to 1/4 to 1/2 length into corpus bursae; corpus bursae oval, signum basal plate rounded, covered with short thorns and two anteriorly directed horn-shaped lobes.

##### Remarks.

The female genitalia of *F.multicornuta* (Figs 48, 49) resemble that of *F.zagulajevi* Anikin & Piskunov, 1996 (Fig. 50), but the left side of the ductus bursae is more densely covered with microspines, whereas the right side is less covered with microspines in *F.zagulajevi*. Additionally, the longitudinal folds are longer and the medial sclerite is shorter in *F.zagulajevi* (Fig. 50).

### ﻿An annotated list of the species of *Filatima* in the Palaearctic region

#### ﻿ Filatima algarbiella

Taxon classificationAnimaliaLepidopteraGelechiidae

Corley, 2014

171B8C5F-21D7-598C-8541-B8C3CF8E8F28

Filatima algarbiella Corley, 2014: 233

##### Distribution.

Portugal (Corley 2014: 233).

#### ﻿ Filatima angustipennis

Taxon classificationAnimaliaLepidopteraGelechiidae

Sattler, 1961

75E10EAB-8A6A-568F-99C6-8FAB1A088F1C

Filatima angustipennis Sattler, 1961: 117
Filatima albicostella auct. (nec Clarke 1942); misidentification

##### Distribution.

France (Sattler 1961: 117), Russia: Altai Republic (Bidzilya 2002: 68).

#### ﻿ Filatima asiatica

Taxon classificationAnimaliaLepidopteraGelechiidae

Sattler, 1961

C88BC238-0480-5032-80F5-A1968EC6E817

Filatima asiatica Sattler, 1961: 119
= Filatimabidentella Bidzilya, 1998. Synonymised by Bidzilya and Nupponen (2018: 392)

##### Distribution.

Kyrgyzstan, Mongolia (Sattler 1961; Piskunov 1979), Russia: Tuva, Buryatia, Zabaikalskiy krai (Bidzilya and Budashkin 1998; Bidzilya and Nupponen 2018).

#### ﻿ Filatima djakovica

Taxon classificationAnimaliaLepidopteraGelechiidae

Anikin & Piskunov, 1996

91C6178F-3F18-5D6A-A96A-1794B86A512E

Filatima djakovica Anikin & Piskunov, 1996: 173

##### Distribution.

Romania (Rákosy et al. 2003), Ukraine (Bidzilya and Budashkin 2017: 13), Russia: Vladimir and Saratov regions (Anikin and Piskunov 1996; Piskunov and Uskov 2006).

#### ﻿ Filatima fontisella

Taxon classificationAnimaliaLepidopteraGelechiidae

Lvovsky & Piskunov, 1989

BC438729-3F5B-549D-9AD5-528801DB436B

Filatima fontisella Lvovsky & Piskunov, 1989: 560

##### Distribution.

Mongolia (Lvovsky and Piskunov 1989: 560).

##### Remarks.

As the only Russian record of *Filatimafontisella* (Kostjuk et al. 1994: 10) is based on misidentification of *F.multicornuta*, this species should be removed from the list of the Lepidoptera of Russia.

#### ﻿ Filatima incomptella

Taxon classificationAnimaliaLepidopteraGelechiidae

(Herrich-Schäffer, 1854)

CFB70CEC-98B9-5682-B4BA-64106E21AA33

[no genus] incomptella Herrich-Schäffer, 1853: pl. 71, fig. 536
Gelechia incomptella Herrich-Schäffer, 1854: 162, 178
= Gelechiaturbidella Nolcken, 1871: 561

##### Distribution.

Europe (Huemer and Karsholt 1999), eastwards to Siberia: Omsk region (Ponomarenko and Knyazev 2020: 281) and Zabaikalskiy krai of Russia.

#### ﻿ Filatima karsholti

Taxon classificationAnimaliaLepidopteraGelechiidae

Ivinskis & Piskunov, 1989

A0C920B0-BD79-505A-B679-39B9AB3C3599

Filatima karsholti Ivinskis & Piskunov, 1989: 572

##### Distribution.

Mongolia, China: Xinjiang (Ivinskis and Piskunov 1989: 575), Russia: Buryatia (Bidzilya and Nupponen 2018: 392).

#### ﻿ Filatima kerzhneri

Taxon classificationAnimaliaLepidopteraGelechiidae

Ivinskis & Piskunov, 1989

BED82E6E-505B-5AF1-85D6-2E7BA18ED1E7

Filatima kerzhneri Ivinskis & Piskunov, 1989: 575

##### Distribution.

Mongolia (Ivinskis and Piskunov 1989: 575).

#### ﻿ Filatima multicornuta

Taxon classificationAnimaliaLepidopteraGelechiidae

Bidzilya & Nupponen, 2018

26BE55AC-31E5-59E4-96EE-6904813BA954

Filatima multicornuta Bidzilya & Nupponen, 2018: 395

##### Distribution.

Mongolia, Russia: Tuva Republic (Bidzilya and Nupponen 2018: 395), Zabaikalskiy krai (new record).

##### New record.

[Russia] • 1 ♂; SE Zabaikalie, Nizhniy Tsasutchei; 4 Aug 1989; [genitalia slide number] 33/17, O. Bidzilya; I. Kostjuk leg.; ZMKU.

#### ﻿ Filatima nigrimediella

Taxon classificationAnimaliaLepidopteraGelechiidae

Bidzilya, 1998

68E8DE96-271D-5FA1-8142-A06698EFBD4C

Filatima nigrimediella Bidzilya, 1998: 53. In Bidzilya et al. 1998: 53.

##### Distribution.

Russia: Zabaikalskiy krai (Bidzilya et al. 1998: 53).

##### Remarks.

The species is known only from the male holotype collected in Borzja, S of Zabaikalskiy kray of Russia. The original description is accompanied by a black and white photograph of the adult and a drawing of male genitalia in lateral view (Bidzilya et al. 1998, Figs 17, 18). Here we provide colour photographs of the holotype (Fig. 44) and the slide of the unrolled male genitalia (Figs 45–47).

#### ﻿ Filatima pagicola

Taxon classificationAnimaliaLepidopteraGelechiidae

(Meyrick, 1936)

5F3A479A-5733-59AC-B32A-B028F8D3B5D2

Gelechia pagicola Meyrick, 1936: 44

##### Distribution.

China: Taishan (Meyrick 1936: 44).

##### Remarks.

The photograph of the lectotype and its male genitalia are illustrated in Clarke (1969: 96, pl. 48, figs 1–1b).

#### ﻿ Filatima pallipalpella

Taxon classificationAnimaliaLepidopteraGelechiidae

(Snellen, 1884)

F38A632E-3FD3-5AA2-8FF9-F712EABAEEFE

Gelechia pallipalpella Snellen, 1884: 167
= Gelechiaautocrossa Meyrick, 1937. In Caradja and Meyrick 1937: 157. Synonymised by Bidzilya and Nupponen (2018: 397)

##### Distribution.

Russia: Lower Volga, southern Ural, Novosibirsk region, Altai, Tuva, South of Krasnoyarskiy krai, Buryatia, Zabaikalskiy krai, Amur region, Primorskiy krai (Ponomarenko 2008, 2016; Junnilainen et al. 2010; Bidzilya and Nupponen 2018: 398), Kyrgyzstan (new record), China: Shandong Province (Caradja and Meyrick 1937: 157).

##### New record.

[Kyrgyzstan] • 1 ♀; Turkestan mts, valley river Kalay-Makhmud; 1830 m; 10 Jun 2010; [DNA barcode identification number] TLMF Lep 21784; N. Pöll leg.; TLMF.

The new record from Kyrgyzstan is based on molecular evidence with barcodes corresponding to samples from S Ural of Russia.

#### ﻿ Filatima sciocrypta

Taxon classificationAnimaliaLepidopteraGelechiidae

(Meyrick, 1936)

4517B5C3-22AE-50E0-B124-C78FE9880C16

Gelechia sciocrypta Meyrick, 1936: 44
= Gelechiadigrapta Meyrick, 1936: 44. Synonymised by Beccaloni et al. (2003)
= Gelechiademophila Meyrick, 1937: 157. In Caradja and Meyrick 1937: 157. Synonymised by Beccaloni et al. (2003)

##### Distribution.

Mongolia (Emelyanov and Piskunov 1982: 393), China: Shandong, Jilin (Meyrick 1936: 44; Caradja and Meyrick 1937: 157); Russia: Buryatia (Bidzilya and Nupponen 2018: 397), Zabaikalskiy krai (Caradja 1938: 92), Amur region (Ponomarenko 2016: 124).

##### Remarks.

The above synonymy is based on NHMUK’s card index and its computerised and updated version (Beccaloni et al. 2003), but it has not been formally published. We did not examine type specimens of *G.digrapta* and *G.demophila* and therefore cannot confirm this synonymy.

#### ﻿ Filatima spurcella

Taxon classificationAnimaliaLepidopteraGelechiidae

(Duponchel, [1843])

2A49A314-DA72-51C3-9B86-C9EB654796B8

Anacampsis spurcella Duponchel, [1843]: 269.
= Gelechiafuscantella Heinemann, 1870: 213

##### Distribution.

Europe, Turkey, Armenia (Sattler 1960: 53; Huemer and Karsholt 1999).

#### ﻿ Filatima tephritidella

Taxon classificationAnimaliaLepidopteraGelechiidae

(Duponchel, 1844)

F0D9FCC7-630C-5C2C-B4EA-602F336CD0F5

Anacampsis tephritidella Duponchel, 1844: 432
Gelechia tephriditella Herrich-Schäffer, 1854: 162, 178,
[no genus] tephriditella Herrich-Schäffer, 1853: pl. 69, figs 517, 518.

##### Distribution.

Europe from France to Lower Volga (Huemer and Karsholt 1999) and western Kazakhstan (Caradja 1920), Omsk region (Ponomarenko and Knyazev 2020: 281) and Tuva Republic of Russia (Bidzilya 2005: 14).

#### ﻿ Filatima textorella

Taxon classificationAnimaliaLepidopteraGelechiidae

(Chrétien, 1908)

C932B900-5CFC-5E48-BCAA-5E2576EC9EB7

Gelechia textorella Chrétien, 1908: 59

##### Distribution.

Spain, France (Huemer and Karsholt 1999), North Macedonia (new record), Turkey (new record).

##### New records.

[North Macedonia] • 1 ♂; Treskaschluht; 1–5 Jun 1967; [genitalia slide number] 85/18, O. Bidzilya; R. Pinker leg.; SMNK; [Turkey] • 1 ♂, 1 ♀; Asia min., Anatolien, Kizilcahamam; 925 m; 3 Jun 1970; [genitalia slide number] 78/18♂. O. Bidzilya; AR0277♀, A.L.M. Rutten; M. and W. Glaser leg.; SMNK.

#### ﻿ Filatima transsilvanella

Taxon classificationAnimaliaLepidopteraGelechiidae

Kovács & Kovács, 2001

7F9AB8AE-4A5E-58F3-9810-D41A7BD38232

Filatima transsilvanella Kovács & Kovács, 2001: 363

##### Distribution.

Romania (Kovács and Kovács, 2001: 363), Russia (South Ural) (Junnilainen et al. 2010: 38).

#### ﻿ Filatima ukrainica

Taxon classificationAnimaliaLepidopteraGelechiidae

Piskunov, 1971

B4CD97F7-7543-54A8-9C07-C0437B8FFF9E

Filatima ukrainica Piskunov, 1971; 1106

##### Distribution.

Ukraine (Piskunov 1971: 1106), Lithuania, Sweden (Ivinskis and Piskunov 1981: 50).

#### ﻿ Filatima zagulajevi

Taxon classificationAnimaliaLepidopteraGelechiidae

Anikin & Piskunov, 1996

88A6D771-B923-562C-AB07-C3F016612BBD

Fig. 50

Filatima zagulajevi Anikin & Piskunov, 1996: 175

##### Distribution.

Russia: Lower Volga, South Ural (Anikin and Piskunov 1996: 175; Piskunov and Anikin 2005: 51; Junnilainen et al. 2010: 39), Kazakhstan (new record).

##### New record.

[Kazakhstan] • 1 ♀; Sopki Kokshetau near Tersakkan river; 4 Jun 1958; [genitalia slide number] 15/18, O. Bidzilya; M. Falkovitsh leg.; ZIN.

## Supplementary Material

XML Treatment for Filatima armata

XML Treatment for Filatima subarmata

XML Treatment for Filatima afghana

XML Treatment for Filatima karii

XML Treatment for Filatima multicornuta

XML Treatment for Filatima algarbiella

XML Treatment for Filatima angustipennis

XML Treatment for Filatima asiatica

XML Treatment for Filatima djakovica

XML Treatment for Filatima fontisella

XML Treatment for Filatima incomptella

XML Treatment for Filatima karsholti

XML Treatment for Filatima kerzhneri

XML Treatment for Filatima multicornuta

XML Treatment for Filatima nigrimediella

XML Treatment for Filatima pagicola

XML Treatment for Filatima pallipalpella

XML Treatment for Filatima sciocrypta

XML Treatment for Filatima spurcella

XML Treatment for Filatima tephritidella

XML Treatment for Filatima textorella

XML Treatment for Filatima transsilvanella

XML Treatment for Filatima ukrainica

XML Treatment for Filatima zagulajevi
